# Transcriptome Alteration in the Diabetic Heart by Rosiglitazone: Implications for Cardiovascular Mortality

**DOI:** 10.1371/journal.pone.0002609

**Published:** 2008-07-09

**Authors:** Kitchener D. Wilson, Zongjin Li, Roger Wagner, Patrick Yue, Phillip Tsao, Gergana Nestorova, Mei Huang, David L. Hirschberg, Paul G. Yock, Thomas Quertermous, Joseph C. Wu

**Affiliations:** 1 Department of Radiology, Stanford University School of Medicine, Stanford, California, United States of America; 2 Department of Medicine, Division of Cardiology, Stanford University School of Medicine, Stanford, California, United States of America; 3 Department of Bioengineering, Stanford University School of Medicine, Stanford, California, United States of America; 4 Human Immune Monitoring Center, Stanford University School of Medicine, Stanford, California, United States of America; Cleveland Clinic Foundation, United States of America

## Abstract

**Background:**

Recently, the type 2 diabetes medication, rosiglitazone, has come under scrutiny for possibly increasing the risk of cardiac disease and death. To investigate the effects of rosiglitazone on the diabetic heart, we performed cardiac transcriptional profiling and imaging studies of a murine model of type 2 diabetes, the C57BL/KLS-lepr^db^/lepr^db^ (*db/db*) mouse.

**Methods and Findings:**

We compared cardiac gene expression profiles from three groups: untreated *db/db* mice, *db/db* mice after rosiglitazone treatment, and non-diabetic *db/+* mice. Prior to sacrifice, we also performed cardiac magnetic resonance (CMR) and echocardiography. As expected, overall the *db/db* gene expression signature was markedly different from control, but to our surprise was not significantly reversed with rosiglitazone. In particular, we have uncovered a number of rosiglitazone modulated genes and pathways that may play a role in the pathophysiology of the increase in cardiac mortality as seen in several recent meta-analyses. Specifically, the cumulative upregulation of (1) a matrix metalloproteinase gene that has previously been implicated in plaque rupture, (2) potassium channel genes involved in membrane potential maintenance and action potential generation, and (3) sphingolipid and ceramide metabolism-related genes, together give cause for concern over rosiglitazone's safety. Lastly, *in vivo* imaging studies revealed minimal differences between rosiglitazone-treated and untreated *db/db* mouse hearts, indicating that rosiglitazone's effects on gene expression in the heart do not immediately turn into detectable gross functional changes.

**Conclusions:**

This study maps the genomic expression patterns in the hearts of the *db/db* murine model of diabetes and illustrates the impact of rosiglitazone on these patterns. The *db/db* gene expression signature was markedly different from control, and was not reversed with rosiglitazone. A smaller number of unique and interesting changes in gene expression were noted with rosiglitazone treatment. Further study of these genes and molecular pathways will provide important insights into the cardiac decompensation associated with both diabetes and rosiglitazone treatment.

## Introduction

Cardiovascular disease is the leading cause of morbidity and mortality in patients with type 2 diabetes [1]. While atherosclerotic coronary artery disease is highly prevalent in many diabetic patients, the occurrence of nonischemic cardiomyopathy (“diabetic cardiomyopathy”) suggests that other processes such as microangiopathy, metabolic factors, or myocardial fibrosis [1] might be involved. Thiazolidinediones (TZDs) such as rosiglitazone are insulin sensitizers that may also have beneficial properties for diabetic cardiomyopathy. These agonists bind to a subfamily of nuclear hormone receptors, the peroxisome proliferator-activated receptors (PPARs, including α, β/δ and γ isoforms, of which the γ isoform is the most common target), and activate transcription factors that modulate gene expression, ultimately leading to increased insulin sensitivity in peripheral tissues through poorly defined pathways [2], [3]. However, a recent meta-analysis of clinical trial outcomes for the PPARγ agonist rosiglitazone found a significant increase in the risk of myocardial infarction, and a borderline significant risk of death from cardiovascular causes [4]. The reasons for this increased risk are unclear, and a better understanding of the effects of rosiglitazone on the diabetic heart is urgently needed.

Despite the large number of studies on rosiglitazone and diabetes, the transcriptional network changes by which PPARγ agonists and other TZDs promote insulin sensitivity are not well characterized. A number of groups have studied gene expression in the *db/db* mouse liver [5], as well as transcriptional changes induced by TZDs in adipocytes, kidney, aorta, and pancreatic islet cells in various mouse models [6], [7], [8], [9], but none has studied the heart directly. Moreover, determining PPAR-agonist target tissues is complicated by the fact that the three PPAR isoforms are not distributed equally across all tissues. In adipose tissue, PPARγ predominates and promotes differentiation and lipid storage [10], resulting in suppression of lipolysis by insulin and reduction of plasma free fatty acid concentrations. In the cardiovascular system, PPARγ is expressed in vascular smooth muscle and vascular endothelium [11], but is believed to be in low abundance in cardiomyocytes [12].

Animal models of type 2 diabetes present an optimal system for studying the effects of rosiglitazone. A well-established murine model is the C57BL/KLS-lepr^db^/lepr^db^ (*db/db*) mouse that has a mutation in the leptin receptor. Homozygous *db/db* mice become obese by 3–4 wk of age and develop hyperglycemia at 4–8 wk. Serum insulin levels increase as early as 10–14 days, peak at 6–8 wk, then decrease afterward. These mice continue to be hyperinsulinemic throughout life and ultimately develop cardiomyopathy (contractile dysfunction), as evidenced from metabolic experiments using cultured *db/db* cardiomyocytes [13], echocardiographic studies [14], isolated working heart preparations [15], and direct *in situ* ventricular pressure measurements [16]. Recent studies by our lab and others have delineated the longitudinal structural and metabolic cardiomyopathic changes in the *db/db* mouse using cardiac magnetic resonance (CMR) and Fluorine-18–2-fluoro-2-deoxy-d-glucose ([^18^F]FDG) PET scanning [17].

To address the many questions concerning the molecular and functional effects of rosiglitazone on the diabetic heart, we used CMR imaging and echocardiagraphic studies to assess the development of cardiomyopathy in untreated and rosiglitazone-treated *db/db* mice, as well as normal *db/+* mice. These studies were followed by cardiac gene expression profiling of the diabetic and control phenotypes to understand the underlying molecular changes in heart tissue. A comparison of the cardiac transcriptomes of rosiglitazone-treated *db/db* mice with untreated *db/db* and normal *db/+* mice thus provides insights into the effects of rosiglitazone on the heart, either through direct or indirect mechanisms, as well as clues to its potential toxicity.

## Methods

### Animal and sample tissue preparation

Homozygous *db/db* mice (Jackson Laboratories, Bar Harbor, ME) were maintained on a normal chow diet and housed in a room with a 12∶12-h light-dark cycle and an ambient temperature of 22°C. For the treatment group, homozygous *db/db* mice were maintained on 5 mg/kg/day (approximately 0.225 mg/mouse/day) rosiglitazone (GlaxoSmithKline, London, UK) that was mixed with normal chow. Unless otherwise stated, heterozygous *db/+* littermates were used as control animals. All protocols were approved by the Administrative Panel on Laboratory Animal Care at Stanford University and were carried out in accordance with the guidelines of the American Association for Accreditation of Laboratory Animal Care. At the end of the study, animals were euthanized with a lethal dose of isoflurane. Immediately after death, wet heart weight (HW) and body weight (BW) were measured. Whole hearts, pancreas, and liver were harvested and preserved in TRIzol reagent (Invitrogen, San Diego, CA) for subsequent mRNA isolation, and a subset of hearts were fixed in 10% formalin for histological evaluation.

### Insulin tolerance testing

Insulin tolerance testing was performed on mice after a 6-h fast. At the time of testing, a bolus of human regular insulin (Eli Lilly, Indianapolis, IN; 0.75 IU/kg) was injected intraperitoneally. In blood derived from a tail nick, glucose levels were then determined with a FreeStyle blood glucose monitoring system (Abbott Laboratories, Abbott Park, IL) at baseline and 30, 60, and 120 min after injection.

### Insulin Enzyme-Linked ImmunoSorbent Assay (ELISA)

After a 6-h fast, roughly 200 µl of blood was obtained from each animal via retroorbital bleeding. Samples were placed in 500- µl tubes containing EDTA (Becton-Dickinson, Franklin Lakes, NJ) and centrifuged at 4°C and 13,200 rpm for 10 min. Approximately 50–75 µl of supernatant (serum) was then collected for further processing. Serum insulin concentrations were measured with a Mercodia mouse insulin enzyme immunoassay kit (Alpco Diagnostics, Salem, NH).

### Nonesterified fatty acids (NEFA)

Plasma nonesterified fatty acid concentrations were measured using a HR series NEFA-HR(2) colorimetric assay kit (Wako Chemicals USA, Richmond, VA).

### Left ventricular functional analysis with echocardiogram

Echocardiography was performed by a blinded investigator (ZL) using the Siemens-Acuson Sequioa C512 system equipped with a multi-frequency (8–14 MHz) 15L8 transducer. Analysis of M-mode images was performed using the Siemens built-in software. Left ventricular end diastolic diameter (EDD) and end-systolic diameter (ESD) were measured and used to calculate fractional shortening (FS) by the following formula: FS = [EDD-ESD]/EDD. LV volume at end diastolic (EDV) and end-systole (ESV) were calculated by the bullet method as follows: EDV = 0.85×CSA(d)×L(d), ESV = 0.85×CSA(s)×L(s), where CSA(d) and (s) are endocardial area in end-diastole and end-systole, respectively, obtained from short-axis view at the level of the papillary muscles. L(d) and L(s) are the LV length (apex to mid-mitral annulus plane) in end-diastole and end-systole, respectively, obtained from the parasternal long-axis view. LV ejection fraction (EF%) was calculated as: EF% = (EDV−ESV)×100/EDV.

### Cardiac magnetic resonance imaging

To prepare for scanning, induction of anesthesia was accomplished with 2% isoflurane and 1 l/min oxygen. Respiratory rate was monitored and used to manually calibrate the maintenance dose of isoflurane at 1.25–1.5%. Platinum needle ECG leads were inserted subcutaneously in the right and left anterior chest wall. Respiration was monitored with a pneumatic pillow sensor positioned along the abdomen. Body temperature was maintained at 36–37°C by a flow of heated air thermostatically controlled by a rectal temperature probe. Heart rate (HR), respiratory rate, and body temperature were recorded every 4 min during image acquisition. Magnetic resonance images were acquired by a blinded investigator (PY) with a 4.7-T magnet (Bruker BioSpin, Fremont, CA) controlled by a Varian Inova Console (Varian, Palo Alto, CA), using a transmit-receive quadrature volume coil with an inner diameter of 3.5 cm. For particularly obese animals, a larger coil with an inner diameter of 6 cm was utilized. Image acquisition was gated to the ECG R-wave (Small Animal Instruments, Stony Brook, NY). Coronal and axial scout images were used to position a two-dimensional imaging plane along the short axis of the left ventricular (LV) cavity. Gated gradient echo sequences were then used to acquire sequential short-axis slices spaced 1 mm apart from apex to base. For each sequence, 12 cine frames encompassing one cardiac cycle were obtained at each slice level with the following sequence parameters: acquisition time (TR) = 100–140 ms, echo time (TE) = 2.8–3.5 ms, number of repeats (NEX) = 8, field of view (FOV) = 30×30 mm, matrix = 128×128, flip angle = 60°. For each short-axis slice, planimetry measurements of LV myocardial area were conducted off-line by tracing the epicardial and endocardial borders at end systole and end diastole with MRVision software (MRVision, Winchester, MA). For these purposes the papillary muscles were considered part of the LV cavity. Anteroseptal and posterior wall thickness measurements were performed on a midventricular slice at the level of the papillary muscles at end diastole. LV mass (LVM) was derived from the sum of the differences between the end-diastolic epicardial and endocardial areas from apex to base, adjusted for the specific gravity of myocardial tissue (1.055 g/ml). LV end-diastolic (LVEDV) and end-systolic (LVESV) volumes were calculated as the sum of the endocardial areas of each slice from the apex to the LV outflow tract at end diastole and end systole, respectively. LV ejection fraction (LVEF) was calculated as (LVEDV−LVESV)/LVEDV. Cardiac output (CO) was calculated as (LVEDV−LVESV)/(HR).

### RNA isolation and quality control

Total RNA was isolated separately from five heart samples for each condition using TRIzol reagent (Invitrogen, San Diego, CA) according to the manufacturer's instructions for a total of 15 distinct RNA samples. Total RNA was purified using RNeasy columns (Qiagen, Chatsworth, CA). RNA concentration was measured by spectrophotometry, and RNA integrity assessed with an Agilent 2100 bioanalyzer (Agilent Technologies, Santa Clara, CA) with 6000 Nano Chips according to the manufacturer's instructions. RNA was judged as suitable for array hybridization only if samples exhibited intact bands corresponding to 18S and 28S ribosomal RNA subunits and had a RNA Integrity Number (RIN) greater than six. Universal Reference RNAs for mouse (Stratagene, La Jolla, CA) were purchased as microarray reference controls.

### Labeling reaction and hybridization

Using Low RNA Input Fluorescent Linear Amplification Kits (Agilent Technologies, Santa Clara, CA, USA), cDNA was reverse transcribed from each RNA sample, and cRNA was then transcribed and fluorescently labeled from each cDNA sample. The fifteen mouse heart tissue cRNA samples were labeled with Cy5 and the Universal Mouse reference cRNA was labeled with Cy3, yielding a total of 5 biological replicates per condition. The resulting cRNA was purified using an RNeasy kit (Qiagen, Valencia, CA, USA) followed by quantification of the cRNA by spectroscopy using an ND-1000 spectrophotometer (NanoDrop Technologies, Wilmington, DE, USA). 825 ng Cy3- and Cy5- labeled and amplified cRNA was mixed and fragmented according to the Agilent technology protocol. cRNA was hybridized to 4×44K whole human genome microarray slides from Agilent (Part G4112F) according to the manufacturer's instructions. The hybridization was carried in a rotating hybridization chamber in the dark at 65°C for 17 h.

### Washing, scanning, and feature extraction

Slides were washed with Gene Expression Wash Buffer 1 and 2 (Agilent Technologies, Santa Clara, CA) followed by Acetonitrile. A final wash in Stabilization and Drying Solution was performed to prevent Cy-5 degradation by ozone. The array was scanned using an Agilent G2505B DNA microarray scanner under extended Dynamic range. The image files were extracted using the Agilent Feature Extraction software version 9.5.1 applying LOWESS background subtraction and dye-normalization. Further analyses were performed with BRB ArrayTools Version 3.4 (National Cancer Institute) and TIGR MeV software (http://www.tm4.org/).

### Real-time quantitative PCR for confirmation of microarray results

RNA to cDNA conversion was performed with a SuperScript First-Strand cDNA synthesis kit (Invitrogen). The cDNA was then used as a template in a TaqMan real-time PCR assay with the ABI Prism 7700 Sequence Detection System (Applied Biosystems, Foster City, CA). All samples were run in triplicates. Specialized premade gene expression assay reagents for potassium channel, subfamily K, member 1 (Kcnk1; catalog no. Mm00434624_m1), matrix metalloproteinase 3 (Mmp3; Mm00440295_m1), beta-1,4-N-acetyl-galactosaminyl transferase 1 (B4galnt1; Mm00484653_m1), carboxyl ester lipase (Cel; Mm00486975_m1), and N-acylsphingosine amidohydrolase 2 (Asah2; Mm00479659_m1), purchased from Applied Biosystems, were used for these experiments. Threshold cycles were placed in the logarithmic portion of the amplification curve, and each sample was referenced to 18S RNA (part no. 4319413E) amplification to control for the total amount of RNA. Fold differences between samples (relative quantification) were calculated with the delta-delta method [S1/S2 = 2^−(T1–T2)^], where S1 and S2 represent samples 1 and 2 and T1 and T2 denote the threshold cycles for S1 and S2.

### Histologic analysis of cardiac tissue

At the time of death, a select group of hearts were fixed in 10% formalin, cut at the midventricular level, and embedded in paraffin blocks. The blocks were then sectioned into short-axis slices, which were subsequently stained with hematoxylin and eoxin according to standard protocols. High-power fields magnified to 40× from the midportion of the LV free wall were photographed.

### Statistical Analysis

For all tests, one-way ANOVA was performed. All *P* values <0.05 were considered significant. For microarray analysis, the Significance Analysis of Microarrays (SAM) algorithm was used to identify genes with statistically significant differences in expression among the conditions. SAM is a statistically rigorous test that incorporates a FDR calculation to correct for multiple testing errors. Gene Ontology group overrepresentation analysis was performed using Fisher's exact test with FDR correction through High Throughput GoMiner software.

## Results

### Insulin resistance in rosiglitazone-treated, untreated and control mice

**Figure 1:**
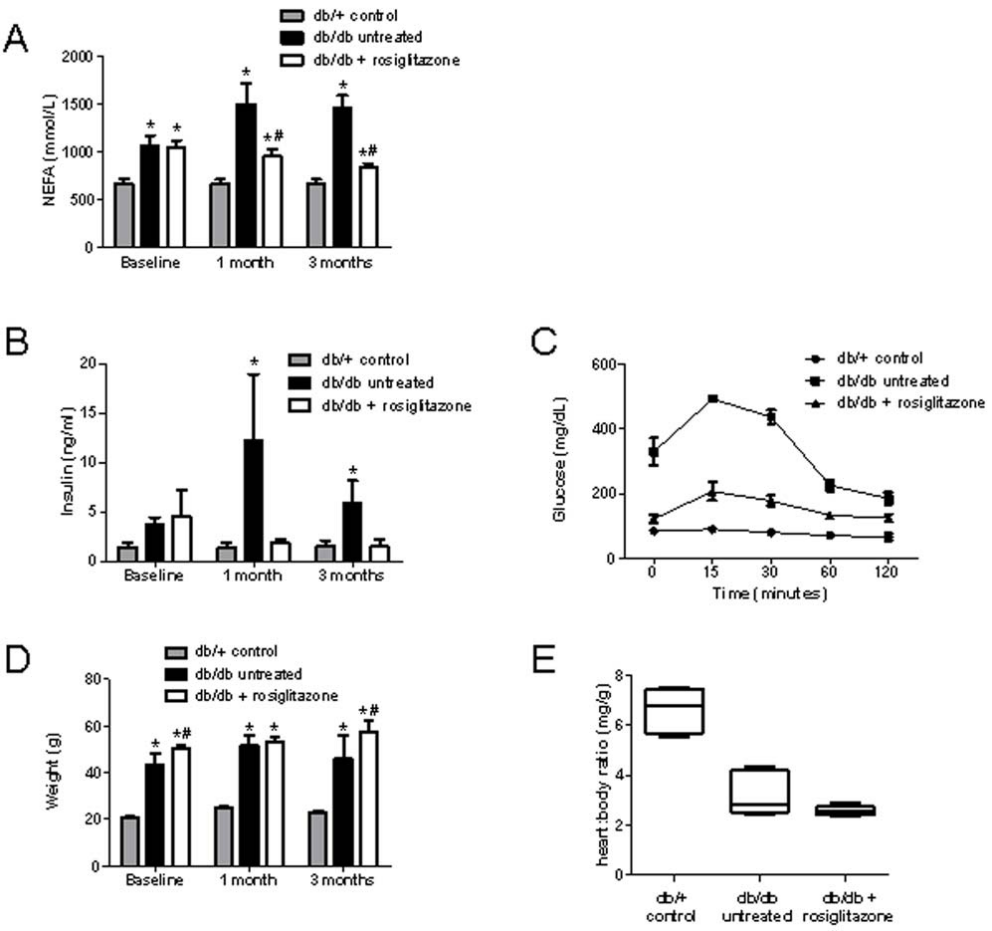
Insulin resistance and mean body/heart weights of rosiglitazone-treated, untreated, and control mice. (A) Plasma NEFA levels at 0, 1, and 3 months in untreated *db/db* mice (n = 10), rosiglitazone-treated *db/db* mice (n = 10), and *db/+* mice (n = 10). NEFA levels were higher in both the treated and untreated *db/db* groups at baseline when compared to *db/+* control mice. Compared to untreated *db/db* mice, NEFA levels in the rosiglitazone-treated *db/db* group decreased significantly during the first month (960.9 vs. 1501.0 mmol/L, *P*<0.05), and decreased further by the third month to near *db/+* control levels. (B) Fasting insulin levels in the three groups at 0, 1, and 3 months. At baseline, both treated and untreated *db/db* groups had increased levels of insulin compared to *db/+* control. Insulin levels dramatically increased in untreated *db/db* mice at 1 month, but progressively decreased in rosiglitazone-treated mice. (C) Insulin tolerance testing at 3 months. Serum glucose levels after insulin injection were significantly higher in untreated *db/db* mice when compared to *db/+* mice; the rosiglitazone-treated *db/db* group had moderately elevated glucose levels after insulin administration. These results corroborate the NEFA and insulin studies that demonstrate improved insulin sensitivity with rosiglitazone treatment. (D) Mean body weights of the three groups of mice at 0, 1, and 3 months. Weights of both treated and untreated *db/db* mice were markedly higher than *db/+* controls at baseline. Furthermore, rosiglitazone-treated mice had generally higher mean body weights when compared to untreated mice. (E) Mean heart/body weight ratios at sacrifice (4 months after treatment initiation). No significant difference between treated and untreated groups (3.27 vs. 2.58, *P* = 0.13), though there does appear to be a downward trend in the data. Heart/body weight ratio was significantly higher in the *db/+* group compared to both *db/db* groups. Values are mean±SEM. *P<0.05 vs. age-matched *db/+*; #*P*<0.05 vs. age-matched untreated *db/db*.

We analyzed a number of metabolic parameters to confirm insulin resistance in our *db/db* murine model of diabetes before proceeding with microarrays. Insulin resistance is strongly associated with obesity, and one mechanism may be the generation of metabolic messengers such as free fatty acids by adipose tissue that inhibit insulin action on muscle [18], [19]. We measured plasma non-esterified fatty acid (NEFA) and insulin levels to follow insulin resistance in our three groups of mice. **Figure 1a** shows plasma NEFA levels in the three groups of mice. Compared to untreated *db/db* mice, NEFA levels in the rosiglitazone-treated *db/db* mice decreased dramatically. Fasting insulin levels (**Figure 1b**) and insulin tolerance testing (**Figure 1c**) further confirmed the improved insulin sensitivity with rosiglitazone treatment.

### Body and heart morphometric analysis

**Figure 2:**
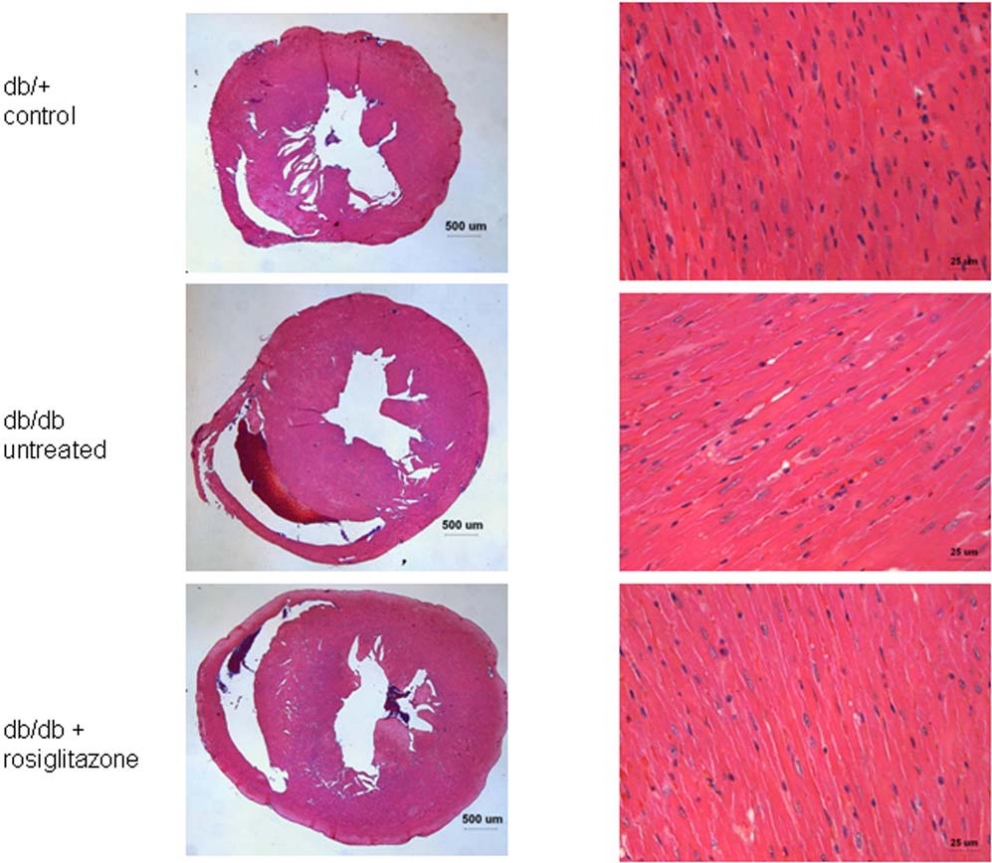
High-power light microscopy slides of myocardial tissue from rosiglitazone-treated and untreated *db/db* mice, and *db/+* controls. Individual cardiomyocytes from all three groups appear qualitatively similar based on morphology and size, though the untreated *db/db* cardiomyocytes exhibit very mild thickening. No increases in inflammatory cells, cell death, fibrosis, or other processes were observed in the two *db/db* groups compared to *db/+* controls.

In addition to the obesity associated with type 2 diabetes, rosiglitazone and other TZDs are widely known to cause further weight gain due to fluid retention and altered adipogenesis [20]. Mean body weights of both treated and untreated *db/db* mice were markedly higher than *db/+* controls at baseline (**Figure 1d**). Furthermore, rosiglitazone-treated mice had generally higher mean body weights when compared to untreated mice, as expected. The heart/body weight ratio was significantly higher in the *db/+* group when compared to the treated and untreated *db/db* groups (**Figure 1e**). However, histological analysis did not reveal obvious differences at the microscopic level (**Figure 2**).

### Left ventricular functional analysis with echocardiogram

**Figure 3:**
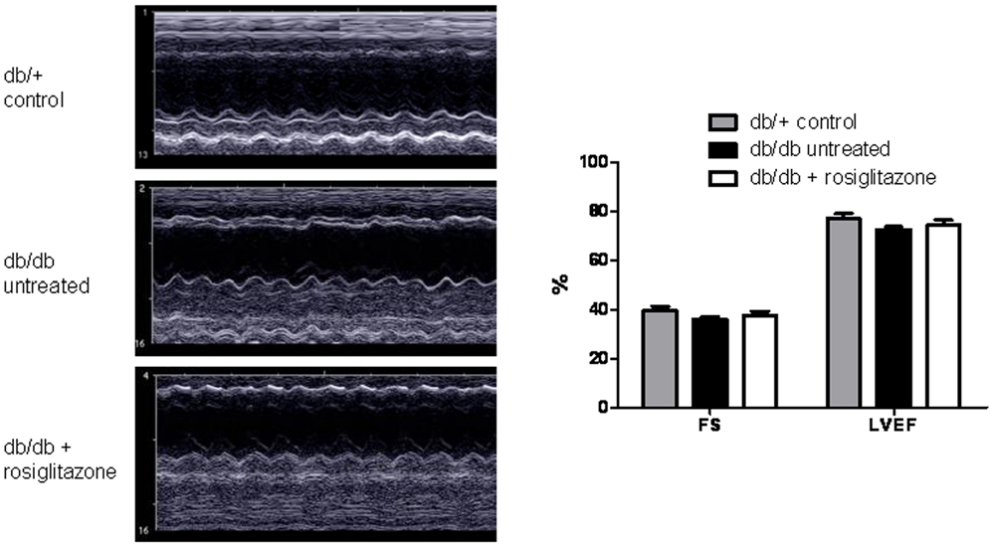
Left ventricular functional analysis with echocardiogram. The mean left ventricular ejection fraction (LVEF) and fractional shortening (FS) of the untreated *db/db* group (72.4±2.3% and 36.2±1.7%, respectively) were not significantly different from the *db/+* group (76.9±4.3%, *P* = 0.11 and 39.7±3.6%, *P* = 0.11, respectively), though there appears to be a downward trend in contractility between these two groups. Further, there were no significant LVEF or FS differences between the rosiglitazone-treated *db/db* group (74.3±3.9% and 37.8±3.1%, respectively) and untreated *db/db* group (*P* = 0.43 and 0.40, respectively). Values are mean±SEM.

Previous groups have observed a significant decrease in left ventricular ejection fraction (LVEF) and fractional shortening (FS) in *db/db* mice when compared with *db/+* controls [14], [21]. In one study, the FS was reduced by as much as 16% at 12 weeks of age [14]. However, more accurate CMR studies of cardiac contractility have not confirmed the dramatic differences seen in the echocardiographic studies, observing only an approximately 2% reduction in LVEF at 22 weeks of age [17]. Furthermore, clinical echocardiographic studies of type 2 diabetic patients treated with rosiglitazone found no significant changes in LVEF after 52 weeks of treatment [22]. To investigate cardiac contractility and verify these previous findings, we assessed cardiac contractility in the three mouse groups using echocardiography (**Figure 3**). The mean LVEF and FS levels of the untreated *db/db* group were not significantly different from those in the *db/+* group, though there does appear to be a downward trend in contractility in the *db/db* group. Further, we found no significant LVEF or FS differences between the rosiglitazone-treated *db/db* group and untreated *db/db* group, confirming the results of the clinical study that found no evidence of rosiglitazone-induced changes in cardiac contractility.

### Cardiac magnetic resonance imaging

**Figure 4:**
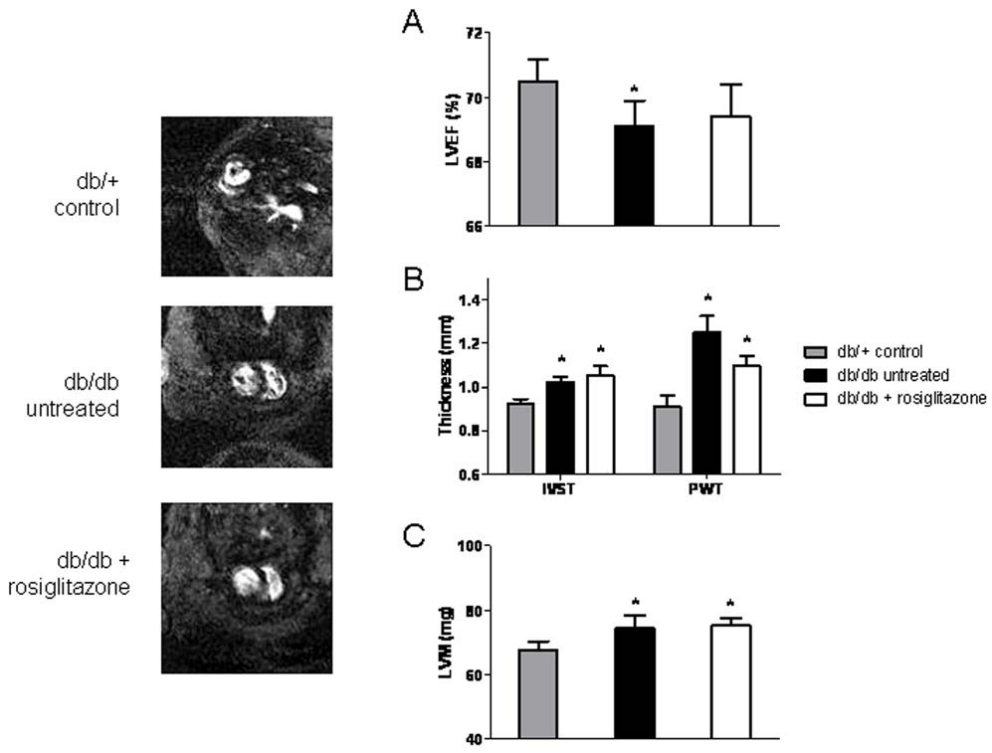
Cardiac magnetic resonance imaging. CMR imaging at 8 weeks after treatment initiation of rosiglitazone-treated *db/db* mice (n = 4), untreated *db/db* mice (n = 4) and *db/+* groups (n = 4). Left ventricular mass (LVM), interventricular septal thickness (IVST) and posterior wall thickness (PWT) were significantly increased in both treated and untreated *db/db* mice relative to *db/+* controls, while LVEF was decreased (though not significantly in the rosiglitazone-treated group). No significant differences in LVM, IVST, PWT, or LVEF between rosiglitazone-treated and untreated *db/db* mice were detected (*P* = 0.82, 0.66, 0.14, and 0.88, respectively). Values are mean±SEM. *P<0.05 vs. age-matched *db/+*.

Using cardiac magnetic resonance (CMR) scanning, our group has previously shown progressive cardiomyopathic changes in *db/db* mice when compared to *db/+* controls [17]. Left ventricular mass (LVM), interventricular septal thickness (IVST) and posterior wall thickness (PWT) were significantly increased in *db/db* mice, while LVEF was only marginally decreased. For the CMR imaging in this new study (**Figure 4**), we included rosiglitazone-treated *db/db* mice in addition to the untreated *db/db* and *db/+* groups, and scanned them at 8 weeks after treatment initiation to look for cardiomyopathic changes. CMR scans revealed significant, albeit subtle, cardiomyopathic changes in untreated *db/db* mice compared to *db/+* controls (**Figure 4**). However, rosiglitazone treatment resulted in no significant improvements in cardiac contractility, which confirmed our echocardiographic studies.

### Gene expression analysis

**Figure 5:**
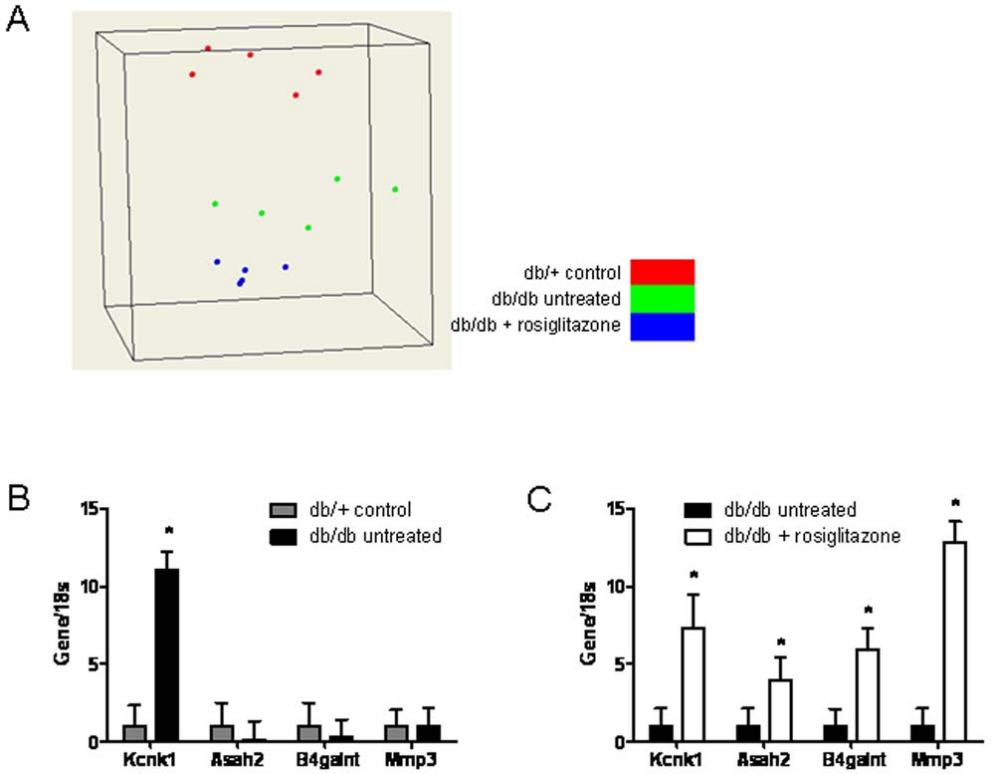
Multidimensional scaling plot of the expression data and real-time PCR analysis. (A) 3-dimensional scaling plot provides a graphical representation of high-dimensional expression data in low dimensions. Each point within a “cloud” represents a single microarray, and the similarity within a set of microarrays is indicated by their physical proximity to one other. As evidenced from the figure, each group (*db/+* control, untreated *db/db*, and rosiglitazone-treated *db/db*) clusters into a distinct grouping, indicating that each group has a similar transcriptome that can be distinguished from the two other groups. Taqman real-time PCR of four selected genes normalized to 18s shows (B) significant upregulation of Kcnk1 in untreated *db/db* vs. *db/+* mice and (C) significant upregulation of all four genes in rosiglitazone-treated vs. untreated *db/db* mice. These data confirm the altered regulatory patterns of these four genes in the microarray data. Values are mean±SEM. *P<0.05 vs. control.

Whole-heart RNA from five mice from each of the three groups after four months with or without treatment was used for microarray analysis. Overall, there were significant transcriptional differences between the *db/+* and both *db/db* groups, while differences between rosiglitazone-treated and untreated *db/db* groups were much less dramatic. **Figure 5a** shows a 3-dimensional scaling plot based on principal component analysis of the expression data, which graphically demonstrates that rosiglitazone did not restore *db/db* transcriptomes to the normal *db/+* state.

Genes that exhibited significant differential expression across the three groups were identified using the SAM statistical algorithm (selected genes are listed in **Tables 1** **,** **2**; full gene txt files are included as **Supplemental Gene Lists S1, S2**). Interesting genes that were significantly upregulated in the untreated *db/db* group compared to control include PPARγ (Pparg, the receptor for rosiglitazone), FK506 binding proteins (Fkbp5 and Fkbp10), several potassium channel proteins (Kcnk1 and Kcnd3), two matrix metalloproteinases (Mmp3 and Mmp8), a cyclin-dependent kinase inhibitor (Cdkn1), and myostatin (Gdf8) (**Table 1**). Specific downregulated genes of interest include the anti-apoptotic gene B-cell lymphoma–leukemia 2 (Bcl2), several tumor necrosis factor-alpha (TNFα) genes and transforming growth factor (TGFβ) genes, vascular endothelial growth factor (Vegfc), adiponectin (Adipoq), and apelin (Apln). We confirmed the microarray results of 4 selected genes with real-time PCR (**Figures 5b, 5c**). When we compared the *db/db* rosiglitazone treated group to control, we found that upregulated genes include the previously-mentioned Mmp3 and Kcnk1 transcripts, as well as a potassium channel-interacting gene (Kcnip4), ryanodine receptor (Ryr1), Myosin IIIB (Myo3b), Patched homolog 1, and apelin (**Table 2**).

**Table 1:** Untreated *db/db* vs. *db/+* control mice.

Gene Symbol	RefSeq ID	UGCluster	Name	Score(d)	Fold Change
Kcnk1	NM_008430	Mm.10800	Potassium channel, subfamily K, member 1	7.82	26.03
Gdf8	NM_010834	-	growth differentiation factor 8 (Gdf8)	5.12	15.93
Egfbp2	NM_010115	-	epidermal growth factor binding protein type B (Egfbp2)	4.77	9.47
Fkbp5	NM_010220	Mm.276405	FK506 binding protein 5	9.76	6.11
Mmp8	NM_008611	Mm.16415	Matrix metallopeptidase 8	5.22	5.93
Edn3	NM_007903	Mm.9478	Endothelin 3	6.59	5.72
Cdkn1a	NM_007669	Mm.195663	Cyclin-dependent kinase inhibitor 1A (P21)	5.13	3.74
Kcnj4	NM_008427	Mm.140760	Potassium inwardly-rectifying channel, subfamily J, member 4	5.06	2.68
Map3k6	NM_016693	Mm.36640	Mitogen-activated protein kinase kinase kinase 6	9.39	2.54
Map3k15	BC031147	Mm.386889	Mitogen-activated protein kinase kinase kinase 15	5.88	2.10
Fkbp10	NM_010221	Mm.3894	FK506 binding protein 10	5.21	2.09
Mmp3	NM_010809	Mm.4993	Matrix metallopeptidase 3	4.30	1.90
Kcnd3	NM_019931	Mm.44530	Potassium voltage-gated channel, Shal-related family, member 3	5.93	1.77
Ryr3	XM_619795	Mm.436657	Ryanodine receptor 3	5.45	1.74
Pparg	NM_011146	Mm.3020	Peroxisome proliferator activated receptor gamma	5.51	1.42
Tnfrsf22	NM_023680	Mm.261384	Tumor necrosis factor receptor superfamily, member 22	4.06	1.40
Aifm2	NM_153779	Mm.286309	Apoptosis-inducing factor, mitochondrion-associated 2	5.62	1.39
Il4ra	NM_001008700	Mm.233802|Mm.441865	interleukin 4 receptor, alpha (Il4ra)	4.96	1.37
Sod1	BC057074	Mm.431677	superoxide dismutase 1, soluble (cDNA clone IMAGE:5697222), partial cds.	4.02	1.34
Adipor2	NM_197985	Mm.291826	Adiponectin receptor 2	4.42	1.22
Myo9a	AK029836	Mm.249545	Myosin IXa	−4.70	−1.26
Casp7	NM_007611	Mm.35687	Caspase 7	−4.46	−1.27
Casp2	NM_007610	Mm.3921|Mm.433648	caspase 2 (Casp2)	−5.10	−1.34
Vegfc	NM_009506	Mm.1402	Vascular endothelial growth factor C	−6.20	−1.39
Il13ra1	NM_133990	Mm.24208	Interleukin 13 receptor, alpha 1	−4.24	−1.40
Gja1	NM_010288	Mm.378921	Gap junction membrane channel protein alpha 1	−4.85	−1.49
Il13ra1	NM_133990	Mm.24208	Interleukin 13 receptor, alpha 1	−4.23	−1.50
Myo1b	NM_010863	Mm.3390	Myosin IB	−6.11	−1.57
Tnfaip8	NM_134131	Mm.27740	Tumor necrosis factor, alpha-induced protein 8	−4.65	−1.71
Adrb2	NM_007420	Mm.5598	Adrenergic receptor, beta 2	−4.91	−1.81
Tnfaip8l1	NM_025566	Mm.2312	Tumor necrosis factor, alpha-induced protein 8-like 1	−5.52	−1.88
Il16	NM_010551	Mm.10137	Interleukin 16	−24.20	−2.42
Myo1d	AK037051	Mm.151948	Myosin ID	−4.19	−2.45
Apln	NM_013912	Mm.29262	Apelin	−4.51	−2.54
Bcl2	NM_009741	257460|Mm.462969	B-cell leukemia/lymphoma 2 (Bcl2), transcript variant 1	−7.36	−2.56
Tnfrsf13c	NM_028075	Mm.240047	Tumor necrosis factor receptor superfamily, member 13c	−6.33	−4.54
Adipoq	NM_009605	Mm.3969	Adiponectin, C1Q and collagen domain containing	−5.31	−4.59

Selected genes that were differentially expressed across untreated *db/db* mice and *db/+* controls. All of the genes presented in the tables have statistically significant expression changes. Listed are the most important parameters, including the standard identifications of the gene, a statistical measure of significance (“Score(d)”), and magnitude of the change (“Fold Change”).

**Table 2:** Rosiglitazone-treated vs. untreated *db/db* mice.

Gene Symbol	RefSeq ID	UGCluster	Name	Score(d)	Fold Change
Kcnip4	NM_030265	Mm.160172	Kv channel interacting protein 4	4.46	4.76
Ptch1	NM_008957	Mm.228798	Patched homolog 1	6.67	3.32
Ryr1	NM_009109	Mm.439745	Ryanodine receptor 1, skeletal muscle	5.27	3.17
Kcnk1	NM_008430	Mm.10800	Potassium channel, subfamily K, member 1	6.38	2.84
Myo3b	AK033795	Mm.99648|Mm.458853	adult male epididymis cDNA, RIKEN full-length enriched library, clone:9230110G05	4.46	2.58
Mmp3	NM_010809	Mm.4993	Matrix metallopeptidase 3	4.70	2.03
Tnfsf18	NM_183391	Mm.276823	Tumor necrosis factor (ligand) superfamily, member 18	3.82	1.86
Apln	NM_013912	Mm.29262	Apelin	3.52	1.80
Anxa13	NM_027211	Mm.237985	Annexin A13	4.04	1.64
St3gal5	NM_011375	Mm.38248	ST3 beta-galactoside alpha-2,3-sialyltransferase 5	5.56	1.62
Col22a1	XM_193814	Mm.322500	Collagen, type XXII, alpha 1	3.65	1.45
Col4a1	NM_009931	Mm.738	Procollagen, type IV, alpha 1	4.98	1.26
Kras	NM_021284	Mm.383182	V-Ki-ras2 Kirsten rat sarcoma viral oncogene homolog	4.40	1.23
B4galnt1	BC022180	Mm.386762	Beta-1,4-N-acetyl-galactosaminyl transferase 1	7.83	1.44
Cel	NM_009885	Mm.236017	Carboxyl ester lipase	6.31	1.38
Pparbp	NM_013634	Mm.12926	Peroxisome proliferator activated receptor binding protein	3.68	1.29
Tnfrsf10b	NM_020275	Mm.193430	Tumor necrosis factor receptor superfamily, member 10b	3.54	1.27
Asah2	NM_018830	Mm.104900	N-acylsphingosine amidohydrolase 2	4.38	1.21
Myh9	NM_022410	Mm.29677	Myosin, heavy polypeptide 9, non-muscle	4.03	1.19
Bdkrb1	NM_007539	Mm.377078	Bradykinin receptor, beta 1	−6.72	−1.96
Hsf1	NM_008296	Mm.347444	Heat shock factor 1	−5.70	−1.17
Col23a1	AK162470	Mm.154093|Mm.392367	12 days embryo embryonic body between diaphragm region and neck cDNA, RIKEN full-length enriched library, clone:9430076L03	−5.70	−1.13

Selected genes that were differentially expressed across rosiglitazone-treated and untreated *db/db* mice.

While identifying individual genes that are differentially regulated in each of the conditions is informative, it is also useful to ascertain which cellular *processes* are up- or down- regulated; therefore, we performed the Gene Ontology pathway overrepresentation analysis using Fisher's exact test. Lipid and protein metabolism, fatty acid beta-oxidation, cell death, apoptosis, peroxisome organization, and biogenesis were significantly upregulated in untreated *db/db* mice when compared to control *db/+* mice (**Supplemental Tables S1, S2, S3, S4**). Among major pathways that were significantly downregulated are cell proliferation and cycle, immune response, blood vessel and vasculature development, and anti-apoptosis. Interestingly, comparing the rosiglitazone-treated *db/db* mice to control mice, we did not see a reversal of the pathways that were upregulated in the untreated *db/db* group. The additional pathways that were significantly upregulated in the rosiglitazone-treated *db/db* hearts, however, included secretion, exocytosis, protein and intracellular transport, cellular protein metabolism, and sphingolipid metabolism.

## Discussion

Few studies have studied the global cardiac gene expression changes resulting from rosiglitazone treatment. Understanding these molecular changes will help elucidate the potential mechanism(s) for the increased cardiac disease observed in diabetic patients treated with this drug. Our microarray results demonstrate that rosiglitazone does not reverse many of the significant gene expression and pathway changes that occur in untreated diabetic hearts, such as apoptosis and lipid metabolism. Furthermore, our microarray analysis indicates that rosiglitazone did not upregulate important insulin-regulated glucose transporters such as Glut4 in the heart, a finding that has been reported previously in isolated rat cardiomyocytes [23]. However, we observed a number of unique genes which appear to be differentially regulated between rosiglitazone-treated and untreated animals that may give mechanistic insights into rosiglitazone's effects on the diabetic heart.

We were initially puzzled to find that PPARγ expression was upregulated in the hearts of untreated diabetic mice, as it has been reported to be low in cardiomyocytes [12], [24], [25]. However, several studies have shown PPARγ expression to be important to cardiac metabolism. One study found that rosiglitazone-treated Zucker fatty rat hearts had improved glucose uptake as well as improved contractility compared to lean rat hearts [19]. Another study found that mice lacking PPARγ in cardiomyocytes exhibited mild cardiac hypertrophy [26]. These findings may reflect an indirect effect of rosiligitazone on the heart, as demonstrated by a study in which metabolic changes in PPARγ-treated *db/db* hearts were thought to be secondary to changes in the supply of exogenous substrates [21]. It appears that cardiac PPARγ expression may play an important though poorly understood role in cardiac physiology, and it is possible that modulation of PPARγ signaling may affect the response to cardiac ischemia.

Matrix metalloproteinases are known to play a critical role in atherosclerosis and cardiovascular tissue remodeling, mediating the balance between matrix accumulation and degradation [27]. We found that rosiglitazone caused significant upregulation of Mmp3, which encodes the matrix metalloproteinase stromelysin. Mmp3 is typically expressed by macrophages within the atherosclerotic plaques found in coronary artery disease [28], and has been shown to promote plaque rupture, myocardial infarction and aneurysm [29]. It is therefore possible that the *in vivo* upregulation of Mmp3 by rosiglitazone will promote plaque rupture, leading to increased rates of myocardial infarction, as well as increasing tissue remodeling after ischemia.

In both untreated and rosiglitazone-treated *db/db* mice, we found several potassium channel-related genes that were highly upregulated, including Kcnip4 and Kcnk1. These findings support the hypothesis that cardiomyocytes from *db/db* hearts exhibit electrophysiological alterations such as attenuated outward K^+^ currents [21], [30]. In general, glycolytic ATP production is important for the normal function of cardiac membrane ion channels and pumps, and inhibition of glycolysis causes altered intracellular ion concentrations that may affect cardiac action potentials. Specifically, Kcnip4 modulates A-type potassium channels and has been shown to increase expression of cardiac A-type Kv-4 channels [31]. Kcnk1 encodes a two-pore potassium channel, TWIK-1, which plays a major role in setting the resting membrane potential in many cell types [32]. Although it is unclear to what degree cardiac arrhythmias might have played a role in the mortality shown in the meta-analysis of rosiglitazone trials [4], the changes we observed in cardiomyocyte potassium channel expression raise some concerns.

Rosiglitazone treatment also upregulated four genes related to sphingolipid metabolism: B4galnt1, Cel, Asah2, and St3gal5. Found in eukaryotic plasma membranes, sphingolipids have emerged as a class of lipid mediators believed to be important for endogenous modulation of cardiovascular function [33], [34]. Generation of sphingolipid metabolites such as ceramide, sphingosine, and sphingosine-1-phosphate can activate smooth muscle cell proliferation, endothelial cell differentiation, apoptosis and cell death, migration, vasoconstriction, and dilation. Specifically, ceramide is an important mediator of lipotoxicity in the heart, and accumulation of this metabolite has been associated with cardiomyocyte apoptosis [35]. Recent evidence indicates that the TZD pioglitazone causes increased *de novo* ceramide synthesis in rat hearts [36], although another group has reported decreased ceramide accumulation and subsequent reduction in apoptosis [37]. Regardless of the specific mechanism involved, our microarray data suggest a significant role for sphingolipid metabolism in the rosiglitazone-treated diabetic heart which we are actively investigating further.

The downregulation of the hormone adiponectin in the diabetic state has been well established in adipocytes and serum [38], but it was not until 2005 that Pineiro *et al.* showed evidence that cardiomyocytes also synthesize adiponectin [39]. Confirming a recent report by Natarajan *et al.* [40], our results demonstrate a reduction of adiponectin in the diabetic heart, though we did not observe restoration of its expression after rosiglitazone treatment. In contrast, the signaling peptide apelin, a recently discovered regulator of cardiac and vascular function [41], was downregulated in the untreated *db/db* group but was restored to normal levels after rosiglitazone treatment. Apelin is expressed in the endothelium of heart, kidney, and lung, and its receptor (APJ) is expressed by myocardial cells and some vascular smooth muscle cells. Apelin has inotropic effects on cardiac contractility and has been shown to decrease systemic vascular resistance, and is therefore likely beneficial during heart failure. In fact, apelin signaling may augment inhibition of the renin-angiotensin system [42], which is known to exacerbate heart failure when left unchecked. Though not well understood, the altered expression of apelin we observed in the diabetic heart in response to rosiglitazone may present a novel pathway for the study of diabetic cardiomyopathy.

In conclusion, this study has mapped the genomic expression patterns in the hearts of the *db/db* murine model of diabetes and the effects of rosiglitazone on these patterns. Overall, as we expected, the *db/db* gene expression signature was markedly different from control, but to our initial surprise was not significantly reversed with rosiglitazone. In fact, many of the transcriptional changes induced by diabetic disease on the heart appeared to be further exacerbated by rosiglitazone. In particular, we have highlighted a number of rosiglitazone modulated genes and pathways that may play a role in the pathophysiology of the increase in cardiac mortality seen in the meta-analysis by Nissen *et al.* The cumulative upregulation of (1) a matrix metalloproteinase gene that has previously been implicated in plaque rupture, (2) potassium channel genes involved in membrane potential maintenance and action potential generation, and (3) sphingolipid and ceramide metabolism related genes, all give cause for concern over rosiglitazone's safety. Interestingly, a second meta-analysis has suggested that pioglitazone, also a TZD, may not have the same negative cardiovascular effects as rosiglitazone [43]. We believe the overall expression changes caused by rosiglitazone in the heart will likely also be found with pioglitazone treatment, though with some differences. This is based on previous microarray studies of adipocytes [44] and hepatocytes [45] that found similar, but not identical, expression profiles after treatment with pioglitazone, rosiglitazone, and other TZDs. Future investigations of how these two drugs differ at the transcriptional level in the heart will further elucidate the networks that govern diabetic cardiovascular function and disease.

## Supporting Information

Table S1 Upregulated Gene Ontology pathways with rosiglitazone-treated db/db mice.
(0.03 MB RTF)

Table S2 Downregulated Gene Ontology pathways with rosiglitazone-treated db/db mice.
(0.02 MB RTF)

Table S3 Upregulated Gene Ontology pathways in untreated db/db mice.
(0.04 MB RTF)

Table S4 Downregulated Gene Ontology pathways in untreated db/db mice.
(0.05 MB RTF)

Gene List S1 txt file of microarray results from untreated db/db vs. db/+ analysis
(0.15 MB TXT)

Gene List S2 txt file of microarray results from db/db+rosiglitazone vs db/db analysis.
(0.04 MB TXT)
